# Study on the Effect of Oxygen Defects on the Electrical and Optical Properties of Thin Films

**DOI:** 10.1155/2018/4592913

**Published:** 2018-04-24

**Authors:** Jin Jeong

**Affiliations:** Department of Physics, Chosun University, 309 Pilmun-daero, Dong-gu, Gwangju 501-759, Republic of Korea

## Abstract

SnO_2_ thin films grown directly on the Si substrate had larger average grain sizes as the power intensity increased, but the average grain size of the SnO_2_ thin films grown in oxygen atmosphere decreased as the power intensity increased. Hall measurement of pure SnO_2_ thin films showed that the carrier density increased with increasing power. However, upon annealing the SnO_2_ thin films, the carrier density decreased with increasing power owing to the formation of oxygen vacancies and the SiO_2_ layer between the Si substrate and SnO_2_ thin films. The photoluminescence (PL) of the SnO_2_ thin film grown in the oxygen atmosphere changed, and it was affected by the oxygen defects at the surface and interfaces of the thin film.

## 1. Introduction

The SnO_2_ thin film has a transparent electrode and has a transparent electrode for a display, a solar cell, a transparent thermal element, and an electric element [1–4]. The SnO_2_ thin film is manufactured by spray, chemical vapor deposition, and sputtering [3, 5–8]. It can be manufactured in large amounts by sputtering, making the products less expensive. However, those manufactured by sputtering have defects caused by variation in temperature, deposition time, amount of oxygen in the chamber, amount of plasma generated (owing to the change in the supply power intensity), and vacuum in the chamber. Jeong et al. reported that the initial state of the substrate when a thin film grows can affect the electrical and optical properties of the thin film [9]. Therefore, in order to improve the electrical and optical properties of the SnO_2_ thin film, it is necessary to secure the reliability of the thin film and to control the defects in the thin film.

## 2. Experimental Details

To manufacture the SnO_2_ thin film by radio frequency (RF) sputtering, the Sn target is installed in the chamber, and power is supplied to the chamber to make plasma, so that the Sn^+^ ions are dropped onto the metal Si substrate in the chamber and simultaneously, the high-purity oxygen (99.99%) is injected to deposit the SnO_2_ thin film. At this time, the amount of Sn + ions to be dropped is closely related to the amount of plasma in the chamber, which is influenced by the power intensity in the chamber. In this experiment, the thin film was grown by changing the deposition temperature in the chamber to 350°C; the amount of oxygen introduced was 40 sccm; the deposition time was 1 h; and the power was changed to 100, 150, and 200 W (S1, S2, and S3). Further, S4, S5, and S6 thin films were deposited on the Si substrate at a temperature of 350°C and an oxygen flow rate of 40 sccm, and then the powers supplied at the same conditions as S1, S2, and S3 films were 100, 150, and 200 W. The experimental conditions are listed in Table 1.

The shapes and average sizes of the thin films were measured by electron microscopy (SEM, Hitachi, S-4700, Japan), and the structural characteristics of the thin films were determined by studying their X-ray diffraction (XRD, Rigaku, Rix-2000, Japan) patterns. The electrical properties of the SnO_2_ thin films were measured using a Hall effect measuring device (HL5500PC, England). The PL of the thin films was measured at room temperature.

## 3. Results and Discussion

**Figure 1:**
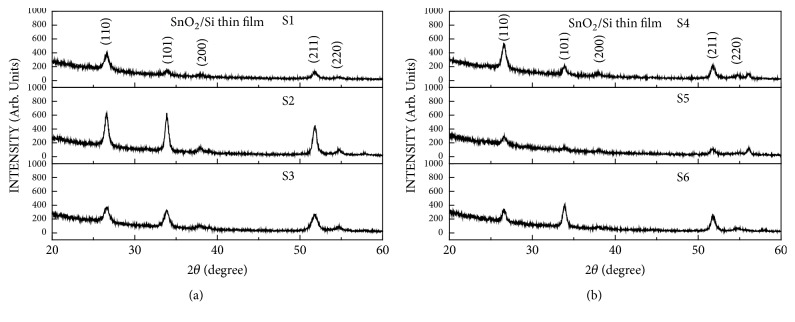
X-ray diffraction patterns of the SnO_2_ thin films of S-1, S-2, and S-3 groups (a) and S-4, S-5, and S-6 groups (b).

Figure 1(a) shows the XRD results of the films grown at a constant power of 100, 150, and 200 W at a deposition temperature of 350°C, a deposition time of 1 h, and an oxygen flow rate of 40 sccm. When the intensity of the supplied power was increased (110), both the (101) plane and the (211) plane had a tendency for crystal growth.

Figure 1(b) shows the XRD results obtained after thin film formation under conditions similar to those for S1, S2, and S3, after oxygen had flowed on the Si substrate for 1 h before the thin film was deposited. The (110), (211), and (101) planes of the thin films increased in intensity as the growth surface decreased. The thin film growth by sputtering is influenced by the power supplied to the chamber. XRD peaks are related to the density of the thin film and the density of the thin film will change as the amount of plasma supplied changes. Figure 1(a) shows that the intensity of the XRD peaks of the thin film is increased and decreased when the power supplied to the chamber is increased to 100, 150, and 200 w. It is seen that the density of the thin film is decreased by decreasing the density of the thin film, which seems to agree with the result of Figure 1(b). Jeong et al. [10, 11] reported that the growth direction of the thin film may change according to the initial growth conditions of thin films. The initial growth condition can be an important parameter in the grain growth of thin films, and changes in the growth surface of the thin film will change the particle shape, particle size, and electrical characteristics of the thin film [12].

**Figure 2:**
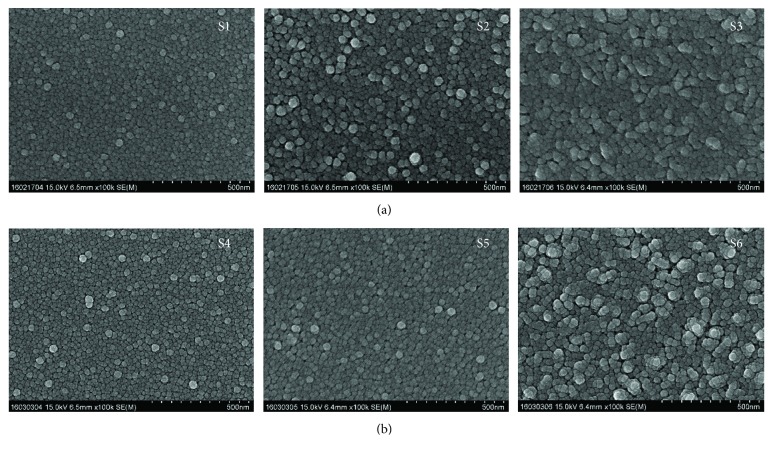
SEM images of the surface of the SnO_2_ thin films of S-1, S-2, and S-3 groups (a) and S-4, S-5, and S-6 groups (b).

Figure 2 shows the surface of S1, S2, and S3 thin films grown at a deposition temperature of 350°C, a deposition time of 1 h, an oxygen flow rate of 40 sccm, and supplied powers of 100, 150, and 200 W. The thin films were circular, and the particle sizes increased with the supplied power. The shape of the thin film and the particle size are correlated with the intensity of the crystal face of the thin film. If the particle size changes, the strength and direction of the growth surface of the thin film may change.

Further, Figure 2(b) shows the SiO_2_ layers deposited by supplying oxygen onto the Si substrate; the thin films were then formed under identical conditions as those for S1, S2, and S3 thin films. The thin films are circular, as in the case of S1, S2, and S3 thin films and the particle size increases with the supplied power. Further understanding of microstructural evolution could be achieved considering that the starting material exhibits structural defects which, as proposed by Cirera et al. [13], are related to high oxygen vacancy concentration.

**Figure 3:**
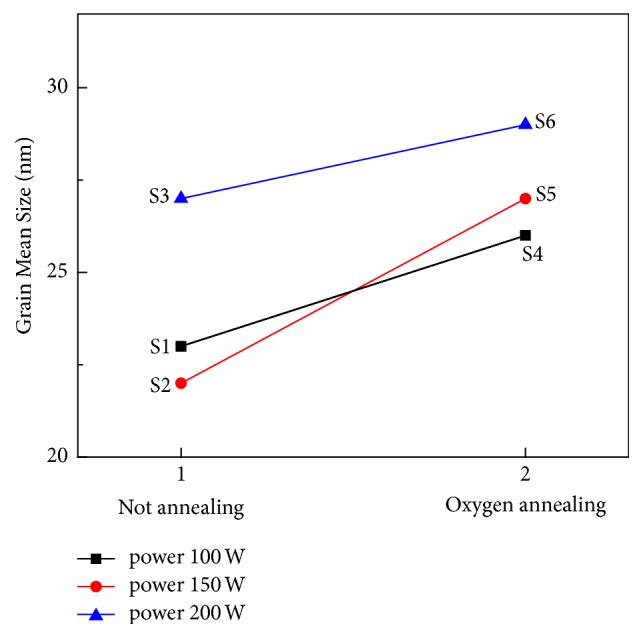
Mean grain size of the SnO_2_ thin films of S-1, S-2, and S-3 groups (1) and S-4, S-5, and S-6 groups (2).

Figure 3 shows the average particle sizes of S1–S6 films. The average particle sizes of S1, S2, and S3 thin films are 23, 22, and 27 nm, respectively. The average particle sizes of S4, S5, and S6 thin films sintered in an oxygen atmosphere are 26, 27, and 29 nm, respectively. The average size spacing of S4, S5, and S6 films sintered in an oxygen atmosphere was less than that of S1–S3 films. When the SnO_2_ thin film is grown after oxygen flows over the Si substrate, the average particle size of the SnO_2_ thin film is influenced by the SiO_2_ layer, and hence, the deviation is reduced even if the power intensity of the particles is increased.

**Figure 4:**
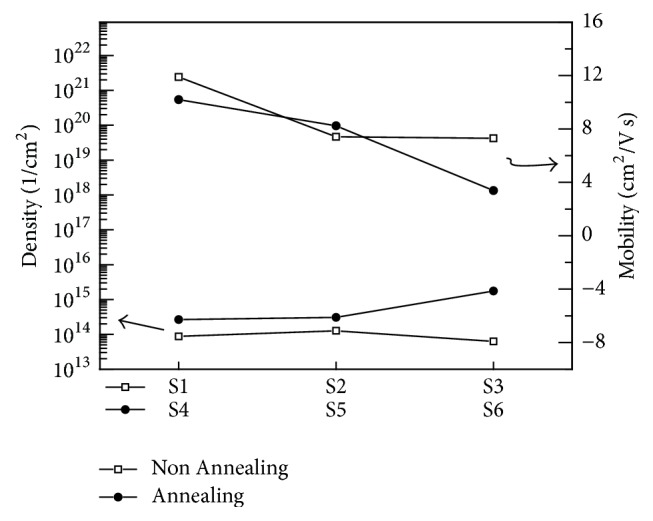
Hall effect data of the SnO_2_ thin films.

**Figure 5:**
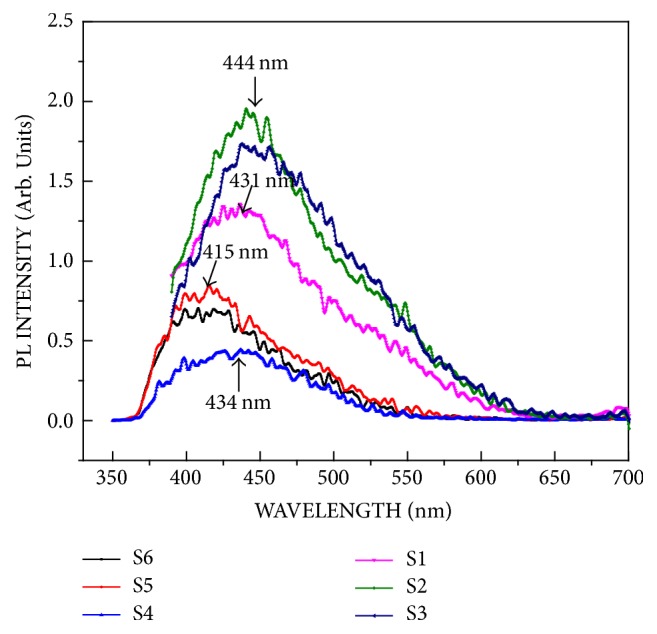
Photoluminescence spectra of the SnO_2_ thin films.

Figure 4 shows the mobility and surface charge density of the thin film obtained through the Hall measurement of thin films S1 to S6; the mobility of S1–S6 was 11.9, 7.41, 7.31, 10.2, 8.24, and 3.39, respectively. The mobility of the thin film decreased as the supplied power increased, and it decreased uniformly when the power supplied to thin films S4, S5, and S6 was increased in the oxygen atmosphere. Thus, the intensity of power supplied to the thin film seems to affect the mobility in the thin film. The surface charge densities of S1, S2, and S3 increased to 8.785 × 10^13^, 1.266 × 10^14^, and 6.246 × 10^13^, respectively. That is, the intensity of power supplied when the thin film was deposited affects the transport charge density of the thin film. The surface charge densities of S4, S5, and S6 films sintered in the oxygen atmosphere were 2.646 × 10^14^, 3.071 × 10^14^, and 1.747 × 10^15^, respectively, and the transport charge density of S4–S6 when the same power as that for S1–S3 was supplied was slightly larger than that of S1–S3. This is because the SiO_2_ + SnO_2_ thin film formed on the Si substrate, and the carrier density increases because of the larger amount of oxygen than that present in the pure SnO_2_ thin film. It was confirmed that the intensity of supplied power affects the surface charge density and the mobility of the thin film and thus that the SiO_2_ layer between the Si substrate and the SnO_2_ thin film affects the electrical properties of the thin film [14]. In general, the fast growth rate of the thin film causes the poor electrical and optical properties due to the increase of roughness and pin hole. In Figures 4 and 5, the decrease in mobility and PL intensity in S3 and S6, which have the largest power during film growth, appears to be due to the poor thin film properties.

Figure 5 shows the PL measurement of thin films S1–S6. The PL intensities of S4, S5, and S6 films with SiO_2_ layer were less than those of S1, S2, and S3 films. In the case of S4, S5, and S6 films, the emission wavelengths of 431 nm and 444 nm (corresponding to S1–S3 films) shifted to 434 nm and 415 nm, respectively. The SnO_2_ thin film grown on the SiO_2_ layer has different oxygen defects on the film surface, and the interface between the substrate and the thin film is different from that between the substrate and pure SnO_2_ thin film: the emission peak shift of the photoluminescence. This is consistent with the change in the carrier density of the surface of the SnO_2_ thin film, as presented in Table 2.

## 4. Conclusion

In the SnO_2_ thin films grown directly on the Si substrate, the intensities of (110) plane, (101) plane, and (211) plane exhibited a strengthening tendency to decrease. However, in SiO_2_+ SnO_2_ thin films, the (110), (101), and (211) planes exhibited a tendency to decrease slowly, and the growth pattern of SnO_2_ thin films changed according to the initial production environment of the thin film. The SEM images of the thin films show that SnO_2_ thin films grown directly on the Si substrate had a large average particle size with increasing power intensity, and the average particle size interval of the SnO_2_ thin films grown on the SiO_2_ layer in the oxygen atmosphere was small. In the Hall effect measurement, the intensity of power supplied to the SnO_2_ thin film was affected by the surface charge density of the thin film, but the thin film deposited by sintering in the oxygen atmosphere showed the surface charge density. It was confirmed that the SiO_2_ layer between the substrate and the thin film affects the electrical properties of the thin film owing to the defects in the SnO_2_ surface and thin film. The optical properties of the thin films grown directly on the substrate were also different from those of the SnO_2_ thin films grown on the SiO_2_ layer by sintering in the oxygen atmosphere.

## Figures and Tables

**Table 1:** Condition of SnO_2_ thin films.

Sample	Power [W]	O flow [sccm]	Deposition temp. [°C]	Deposition time [hr]	Preoxygen time [hr]	Thickness (nm)	Grain size (nm)
S1	100	40	350	1		10	23
S2	150	40	350	1		25	22
S3	200	40	350	1		40	27
S4	100	40	350	1	1	12	26
S5	150	40	350	1	1	30	27
S6	200	40	350	1	1	50	29

**Table 2:** Hall coefficient.

Sample	Resistivity (ohm cm)	Carrier density (cm^−2^)	Mobility (cm^2^/V-s)
S1	0.5984 × 10^4^	8.785 × 10^13^	11.9
S2	0.6651 × 10^4^	1.266 × 10^14^	7.41
S3	1.368 × 10^4^	6.246 × 10^13^	7.31
S4	0.2310 × 10^4^	2.646 × 10^14^	10.2
S5	0.2467 × 10^4^	3.071 × 10^14^	8.24
S6	0.1054 × 10^4^	1.747 × 10^15^	3.39
